# Shoulder adhesive capsulitis: can clinical data correlate with fat-suppressed T2 weighted MRI findings?

**DOI:** 10.1186/s43055-022-00751-x

**Published:** 2022-03-29

**Authors:** Mariam Hussein Mohammed, Fady Micheal Fahmi, Khaled Ali Shehata, Remon Zaher Elia

**Affiliations:** 1 Radiodiagnosis Department, Faculty of Medicine, Ain Shams University, Cairo, Egypt; 2 Orthopedic Surgery Department, Faculty of Medicine, Ain Shams University, Cairo, Egypt

**Keywords:** Adhesive capsulitis of the shoulder, Fat-suppressed T2-weighted MRI

## Abstract

**Background:**

Adhesive capsulitis (AC) of the shoulder or frozen shoulder is a debilitating condition characterized by progressive pain, stiffness and limited range of motion about the glenohumeral joint, the term (adhesive capsulitis) to describe the findings of chronic inflammation and fibrosis of the joint capsule, characterized by significant restriction of both active and passive shoulder motion that occurs in the absence of a known intrinsic shoulder disorder. The goal of the trial was to examine the association between clinical features (stages) and magnetic resonance imaging (MRI) findings in adhesive capsulitis of the shoulder.

**Results:**

In our study, Regarding the diagnostic performance of shoulder MRI there is a statistically significant difference between degree of pain intensity according to Capsular thickening regarding humeral “mm” with *p*-value (*p* < 0.05). The highest value was found moderate and severe pain group (5.67 ± 1.39) compared to absent, minimal and mild pain group (4.42 ± 1.29), there was a statistically significant difference between two groups according to axillary recess regarding height “mm” with *p*-value (*p* < 0.05). The highest value was found absent, minimal and mild pain group (7.02 ± 2.68) compared to moderate and severe pain group (5.73 ± 2.25). There is no statistically significant association between pain intensity and MRI finding regarding capsular edema, extra capsular edema, obliteration of subcoracoid fat triangle, effusion of biceps tendon sheath, capsular thickening of glenoid (mm) and humeral portions of axillary recess and axillary recess width and height (mm), with *p*-value (*p* > 0.05 NS).

**Conclusions:**

MRI is useful for assessing clinical impairment and predicting the clinical stage of adhesive capsulitis***.***

## Background

Adhesive capsulitis of the shoulder or frozen shoulder is a debilitating condition characterized by progressive pain, stiffness and limited range of motion about the glenohumeral joint, the term (adhesive capsulitis) to describe the findings of chronic inflammation and fibrosis of the joint capsule, characterized by significant restriction of both active and passive shoulder motion that occurs in the absence of a known intrinsic shoulder disorder [1].

It is a condition that typically affects about (2–5%) of the general population most commonly middle-aged women (40sy), with some evidence for an association with endocrinological, rheumatological, and autoimmune disease states [2].

Patients with AC usually present with progressively worsening shoulder pain over months followed by significant limitation in shoulder motion. Frequently, there is a significant reduction in the active and passive range of motion in 2 or more planes of motion compared to the unaffected side. Usually, range of motion is lost in the following order: external rotation, abduction, internal rotation, forward flexion [3].

MRI has, in recent years, allowed for the visualization of several characteristic signs seen with this condition, including thickening and shortening of the coracohumeral (CHL) ligament, thus limiting external rotation is due to fibroblastic proliferation within the CHL, thickening of axillary pouch and rotator interval joint capsule, in addition to the obliteration of the subcoracoid fat triangle [4].

The diagnosis is by clinical criteria. There are four clinical stages of adhesive capsulitis (stage I or acute stage): Painful shoulder with mild or moderate reduction of ROM; less than 3 months, stage II (Freezing or subacute) with severe pain and limited ROM from 3 to 9 months, stage III (Frozen or subacute to chronic): stiffness is predominant and pain may be present, from 9 to 14 months, stage IV (Thawing or chronic or fibrotic): Minimal pain with gradual improvement of ROM from 15 to 24 months).

The goal of the trial was to examine the association between clinical features (stages) and MRI findings in adhesive capsulitis of the shoulder.

## Methods

A diagnostic analytical prospective study, we prospectively analyzed the medical charts of 70 cases with 78 shoulders with adhesive capsulitis were included. The study is approved by the ethics review board of our institution. All patients provided informed consent for the MR imaging examinations and for inclusion of their data in this analysis. The MRI findings were correlated with pain intensity, range of motion, and clinical stage. Joint capsule edema in the axillary recess, extracapsular edema, obliteration of the subcoracoid fat triangle, and effusion in the long head biceps tendon sheath were assessed by two radiologists using fat-suppressed T2-weighted images. Joint capsule thickness in the axillary recess and degree of external rotation during MRI were also measured. Associations between MRI findings and clinical features were assessed by statistical analyses.

From September 2020 to March 2021, seventy cases were included in our study group. Their age ranged from 22 to 62 years. With the mean value of the age was 44.84 ± 10.65 years. The study included cases who presented with confirmed (by clinical symptoms, history and full clinical examination) or suspected adhesive capsulitis of the shoulder. The diagnosis is based on MRI findings and clinical criteria. Clinical criteria for the diagnosis of adhesive capsulitis included restricted passive motion of greater than 30 degree in two or more planes of movement, gradually increasing shoulder pain that was more severe at rest for at least 1 month, and normal radiographic findings.

The inclusion criteria of the study were patients who complain from shoulder pain and/or restricted ROM unilaterally or bilaterally with detailed clinical information and history which permit the clinical staging of AC. The initial diagnosis was based on the history and clinical symptoms) clinical criteria for the diagnosis of AC includes: restricted passive motion for greater than 30 degrees in the two or more planes of movement, gradually increasing shoulder pain that was more severe at rest at least 1 month duration, and normal radiographic findings. Patients who fulfilled these criteria underwent shoulder MRI to correlate MRI findings with pain intensity, range of motion and clinical stage.

While, subject refuse to participate in the research and subject with contraindication to MRI as having any non-compatible MRI metallic implants as pacemaker, aneurysm clips, joint replacement or any other electronic or magnetically activated implants as well as claustrophobic subjects were excluded. Also patients with Previous shoulder surgery, substantial shoulder trauma (fracture, dislocation, or extensive soft tissue injury), torn rotator cuff tendon, calcific tendinitis, rheumatoid arthritis, septic arthritis, osteoarthritis, labral lesion, neoplastic condition, neurologic deficit or insufficient medical records were excluded from the study. As described in previous reports, 18 patients with underlying disease (10 with diabetes mellitus, 5 with thyroid disease, one with autoimmune disease, and two with myocardial infarction) were included in this study. Therefore, 78 shoulders in 70 patients (44 women, 26 men). Among the 170 patients who fulfilled the criteria, 100were excluded for the previous reasons and exclusion criteria. Only eight patients out of seventy had bilateral painful shoulders and 62 patients had unilateral shoulder affected.

The study approved from The Ethical Committee of our institution. Written consent was taken from all participates before recruitment in the study after explanation of the purpose and procedures of the study.

Patients were subjected to the following: data collection from subjects or their relatives including careful history taking in the form of full history including the (age, history of any systemic disease, history of trauma) and full clinical examination prior to scanning. MRI scanning of shoulder by a 1.5 T Philips Healthcare, MRI machine at our institution with a dedicated shoulder surface phased –array coil. During imaging, patients were in supine position with no specific preparation of the patients, such as fasting or not drinking, was needed before MRI. Two musculoskeletal radiologists with 15and 10 years of experience who were blinded to the clinical information independently evaluated all of the variables on the MR images.

The following MRI pulse sequences were included: Fat-suppressed T2: coronal oblique, sagittal and axial weighted images, proton density (PD), fast spin echo T1-Weighted sagittal images, time of examination: ranged from 30 to 45 min.

### Statistical analysis

Recorded data were analyzed using the statistical package for social sciences, version 23.0 (SPSS Inc., Chicago, Illinois, USA). Quantitative data were expressed as mean ± standard deviation (SD). Qualitative data were expressed as frequency and percentage, and the confidence interval was set to 95% and the margin of error accepted was set to 5%. So, the *p*-value was considered significant.

## Results

The study was conducted on a wide age group ranging from 22 to 62 years, (mean age of 44.84 ± 10.65 years). There was female predominance with female to male ratio about 1.7:1 (Table 1).

**Table 1:** Clinical symptoms distribution among study group (*n* = 70)

Clinical symptoms	Total (*n* = 70)
*Pain*	
Absent	6 (8.6%)
Minimal	7 (10.0%)
Mild	15 (21.4%)
Moderate	23 (32.9%)
Severe	19 (27.1%)
*Pain scale*	
Range	0–10
Mean ± SD	4.59 ± 2.65
*Limitation of ROM (Abduction)*	
Absent	41 (58.6%)
Present	29 (41.4%)
*Limitation of ROM (External rotation)*	
Absent	19 (27.1%)
Present	51 (72.9%)
*Duration of symptoms (months)*	
Range	0.08–7
Mean ± SD	0.86 ± 1.36
*Side of shoulder*	
Right	35 (50.0%)
Left	35 (50.0%)

There were 6 patients (8.6%) without pain, minimal pain: 7 patients (10.0%), mild pain: 15 patients (21.4%), moderate 23 patients (32.9%) and severe 19 patients (27.1%) among pain intensity, it was ranged 0–10 with mean 4.59 ± 2.65; while limitation of ROM (abduction) 29 patients (41.4%) and limitation of ROM (external rotation) 51 patients (72.9%); as for the right side of shoulder 35 patients (50.0%) and left side of shoulder 35 patients (50.0%), also ranged of duration of symptoms “months” 0.08–7 with mean 0.86 ± 1.36 among clinical symptoms (Table 2).

**Table 2:** Clinical stage distribution among study group (*n* = 70)

Clinical stage	No.	%
*Stage*		
Stage 1	17	24.3
Stage 2	27	38.6
Stage 3	21	30.0
Stage 4	5	7.1
*Type*		
Acute	38	54.3
Acute to subacute	4	5.7
Chronic	10	14.3
Subacute	5	7.1
Subacute to chronic	13	18.6

There were 17 patients (24.3%) in stage 1, 27 patients (38.6%) were in stage 2, 21 patients (30.0%) were in stage 3 and 5 patients (7.1%) were in stage 4 among clinical stage; while according to the type, there were 38 patients (54.3%) in acute stage, 4 patients (5.7%) were in acute to subacute, 10 patients (14.3%) were in chronic, 5 patients (7.1%) were in subacute and 13 patients (18.6%) were in subacute to chronic among clinical stage (Table 3).

**Table 3:** Qualitative findings distribution among study group (*n* = 70)

Qualitative findings	No.	%
*Capsular edema*		
*Glenoid*		
Absent	43	61.4
Present	27	38.6
*Humeral*		
Absent	39	55.7
Present	31	44.3
*Extra capsular edema*		
*Anterior*		
Absent	45	64.3
Present	25	35.7
*Posterior*		
Absent	49	70.0
Present	21	30.0
*Obliteration of subcoracoid fat triangle*		
Absent	35	50.0
Partial obliteration	6	8.6
Complete obliteration	29	41.4
*Effusion of biceps tendon sheath*		
Absent	22	31.4
Present	48	68.6

There were 27 patients (38.6%) had glenoid, 31 patients (44.3%) had humeral among capsular edema, while there were 25 patients (35.7%) had anterior and 21 patients (30.0%) had posterior among extra capsular edema; as for the obliteration of subcoracoid fat triangle 35 patients (50%) and 48 patients (68.6%) had effusion of biceps tendon sheath and it was ranged from 2 to 11 and mean was [4.00 ± 1.83] among qualitative findings.

Table 4 shows that according to quantitative findings, it was mean of glenoid (mm) (5.85 ± 1.28) and mean of humeral (mm) (5.11 ± 1.79) among capsular thickening; while mean of height (mm) (6.75 ± 2.53) and mean of width (mm) (2.49 ± 1.40) among axillary recess.

**Table 4:** Quantitative findings distribution among study group (*n* = 70)

Quantitative findings	Range	Mean ± SD
*Capsular thickening*		
Glenoid (mm)	2.87–8	5.85 ± 1.28
Humeral (mm)	1.5–9.03	5.11 ± 1.79
*Axillary recess*		
Height (mm)	3.2–13.5	6.75 ± 2.53
Width (mm)	0.9–6.8	2.49 ± 1.40

N.B: a case has an inseperable two portions (glenoid and humeral) of capsule as thickened capsule measuring 6 mm and fibrotic axillary recess (chronic stage) as the height can not be measured and the width was lost.

Table 5 shows a statistically significant difference between degree of pain intensity according to capsular thickening regarding humeral porion thickness “mm” with *p*-value (*p* < 0.05). The highest value was found for the moderate and severe pain group (5.67 ± 1.39) compared to minimal and mild pain group (4.42 ± 1.29).

**Table 5:** Association between levels of pain intensity according to MRI finding (n = 70)

MRI finding	Pain intensity		Test value	*p*-value
	Absent, minimal + mild pain (*n* = 28)	Moderate and severe pain (*n* = 42)		
*Qualitative findings*				
*Capsular edema*				
*Glenoid*				
Absent	18 (64.3%)	25 (59.5%)	*x*^2^: 0.161	0.688
Present	10 (35.7%)	17 (40.5%)		
*Humeral*				
Absent	18 (64.3%)	21 (50.0%)	*x*^2^: 1.390	0.238
Present	10 (35.7%)	21 (50.0%)		
*Extra capsular edema*				
*Anterior*				
Absent	18 (64.3%)	27 (64.3%)	*x*^2^: 0.000	1.000
Present	10 (35.7%)	15 (35.7%)		
*Posterior*				
Absent	18 (64.3%)	31 (73.8%)	*x*^2^: 0.726	0.394
Present	10 (35.7%)	11 (26.2%)		
*Obliteration of subcoracoid fat triangle*				
Absent	13 (46.4%)	22 (52.4%)	FE: 0.512	0.774
Partial obliteration	2 (7.1%)	4 (9.5%)		
Complete obliteration	13 (46.4%)	16 (38.1%)		
*Effusion of biceps tendon sheath*				
Absent	10 (35.7%)	12 (28.6%)	*x*^2^: 0.398	0.528
Present	18 (64.3%)	30 (71.4%)		
*Quantitative findings*				
*Capsular thickening*				
*Glenoid (mm)*				
Mean ± SD	6.14 ± 1.03	5.67 ± 1.39	*t*: 1.831	0.130
Range	4–77.6	2.87–8		
*Humeral (mm)*				
Mean ± SD	4.42 ± 1.29	5.30 ± 2.04	*t*: 2.026	0.047*
Range	1.5–7	2–9.03		
*Axillary recess*				
*Height (mm)*				
Mean ± SD	7.02 ± 2.68	5.73 ± 2.25	*t*: 2.176	0.033*
Range	3.49–13.5	3.2–10		
*Width (mm)*				
Mean ± SD	2.45 ± 1.35	2.51 ± 1.45	*t*: 0.040	0.850
Range	1.1–5.5	0.9–6.8		

Using: Independent sample *t*-test; *x*^2^: Chi-square test; Fisher’s exact test

*p*-value > 0.05 NS; *p-value < 0.05 S

Additionally, there was a statistically significant difference between two groups according to axillary recess height “mm” with *p*-value (*p* < 0.05). The highest value was found minimal and mild pain group (7.02 ± 2.68) compared to moderate and severe pain group (5.73 ± 2.25).

There is no statistically significant association between pain intensity and MRI finding regarding capsular edema, extra capsular edema, obliteration of subcoracoid fat triangle, effusion of biceps tendon sheath, capsular thickening glenoid (mm) and axillary recess width (mm), with *p*-value (*p* > 0.05 NS).

Table 6 shows a highly statistically significant difference between absence and presence of limitation of ROM “abduction” according to the thickening of glenoid portion of axillary recess (mm) with *p*-value (*p* < 0.001). The highest value was found for the absent limitation of abduction patient group (6.29 ± 0.99) compared to the present limitation of abduction group (5.21 ± 1.39).

**Table 6:** Association between limitation of ROM “abduction” according to MRI finding (*n* = 70)

MRI finding	Limitation of ROM (Abduction)		Test value	*p*-value
	Absent (*n* = 41)	Present (*n* = 29)		
*Qualitative findings*				
*Capsular edema*				
*Glenoid*				
Absent	23 (56.1%)	20 (69.0%)	*x*^2^: 1.187	0.276
Present	18 (43.9%)	9 (31.0%)		
*Humeral*				
Absent	21 (51.2%)	18 (62.1%)	*x*^2^: 0.810	0.368
Present	20 (48.8%)	11 (37.9%)		
*Extra capsular edema*				
*Anterior*				
Absent	21 (51.2%)	24 (82.8%)	*x*^2^: 6.050	0.014*
Present	20 (48.8%)	5 (17.2%)		
*Posterior*				
Absent	27 (65.9%)	22 (75.9%)	*x*^2^: 0.810	0.368
Present	14 (34.1%)	7 (24.1%)		
*Obliteration of subcoracoid fat triangle*				
Absent	24 (58.5%)	11 (37.9%)	FE: 3.862	0.145
Partial obliteration	4 (9.8%)	2 (6.9%)		
Complete obliteration	13 (31.7%)	16 (55.2%)		
*Effusion of biceps tendon sheath*				
Absent	12 (29.3%)	10 (34.5%)	*x*^2^: 0.214	0.643
Present	29 (70.7%)	19 (65.5%)		
*Quantitative findings*				
*Capsular thickening*				
*Glenoid (mm)*				
Mean ± SD	6.29 ± 0.99	5.21 ± 1.39	*t*: 4.110	< 0.001**
Range	3.9–8	2.87–7.6		
*Humeral (mm)*				
Mean ± SD	4.99 ± 1.75	5.29 ± 1.86	*t*: 0.460	0.500
Range	2–8.8	1.5–9.03		
*Axillary recess*				
*Height (mm)*				
Mean ± SD	7.23 ± 2.56	6.06 ± 2.35	*t*: 1.940	0.060
Range	3.49–13.5	3.2–10.6		
*Width (mm)*				
Mean ± SD	2.35 ± 1.23	2.69 ± 1.62	*t*: 1.010	0.320
Range	0.9–5.5	1.1–6.8		

Using: Independent sample *t*-test; *x*^2^: Chi-square test; Fisher’s exact test

*p*-value > 0.05 NS; *p-value < 0.05 S

Additionally, the results showed 25 patients out of 70 having extra capsular edema anterior, 20 patients (48.8%) belong to the group of patients with absent limitation of abduction and 5 patients (17.2%) belong to the group of patients with present limitation of abduction, as there was a statistically significant negative relation with *p*-value (*p* = 0.014).

There is no statistically significant association between absence and presence of limitation of ROM “abduction” and MRI finding regarding capsular edema of glenoid portion, capsular edema of humeral, posterior extra capsular edema, obliteration of subcoracoid fat triangle, effusion of biceps tendon sheath, capsular thickening of humeral portion (mm), axillary recess height (mm) and axillary recess width (mm), with *p*-value (*p* > 0.05 NS) (Table 7).

**Table 7:** Association between limitation of ROM “external-rotation” according to MRI finding (*n* = 70)

MRI finding	Limitation of ROM (External rotation)		Test value	*p*-value
	Absent (*n* = 19)	Present (*n* = 51)		
*Qualitative findings*				
*Capsular edema*				
*Glenoid*				
Absent	6 (31.6%)	37 (72.5%)	*x*^2^: 9.807	0.002*
Present	13 (68.4%)	14 (27.5%)		
*Humeral*				
Absent	6 (31.6%)	33 (64.7%)	*x*^2^: 6.157	0.013*
Present	13 (68.4%)	18 (35.3%)		
*Extra capsular edema*				
*Anterior*				
Absent	6 (31.6%)	39 (76.5%)	*x*^2^: 12.151	< 0.001**
Present	13 (68.4%)	12 (23.5%)		
*Posterior*				
Absent	8 (42.1%)	41 (80.4%)	*x*^2^: 9.663	0.002*
Present	11 (57.9%)	10 (19.6%)		
*Obliteration of subcoracoid fat triangle*				
Absent	12 (63.2%)	23 (45.1%)	*x*^2^: 3.271	0.195
Partial obliteration	0 (0.0%)	6 (11.8%)		
Complete obliteration	7 (36.8%)	22 (43.1%)		
*Effusion of biceps tendon sheath*				
Absent	7 (36.8%)	15 (29.4%)	*x*^2^: 0.355	0.552
Present	12 (63.2%)	36 (70.6%)		
*Quantitative findings*				
*Capsular thickening*				
*Glenoid (mm)*				
Mean ± SD	6.33 ± 1.15	5.67 ± 1.29	*t*: 1.770	0.060
Range	4–8	2.87–7.7		
*Humeral (mm)*				
Mean ± SD	5.33 ± 1.91	5.03 ± 1.75	*t*: 0.400	0.530
Range	3.2–8.8	1.5–9.03		
*Axillary recess*				
*Height (mm)*				
Mean ± SD	7.05 ± 3.15	6.64 ± 2.27	*t*: 0.370	0.550
Range	3.49–11.6	3.2–13.5		
*Width (mm)*				
Mean ± SD	2.64 ± 1.50	2.43 ± 1.37	*t*: 0.290	0.590
Range	1–5.5	0.9–6.8		

Using: Independent sample *t*-test; *x*^2^: Chi-square test

*p*-value > 0.05 NS; *p-value < 0.05 S

The results showed 27 patients out of 70 having capsular edema of glenoid portion, 13 patients (68.4%) belong to absent limitation of external rotation group and 14 patients (27.5%) belong to present limitation of external rotation group, as there was a statistically significant negative relation with *p*-value (*p* = 0.002).

Additionally, the results showed 31 patients out of 70 having capsular edema of humeral portion, 13 patients (68.4%) belong to absent limitation of external rotation group and 18 patients (35.3%) belong to present limitation of external rotation group, as there was a statistically significant negative relation with *p*-value (*p* = 0.013).

As well as, the results showed 25 patients out of 70 having anterior extra capsular edema, 13 patients (68.4%) belong to absent limitation of external rotation group and 12 patients (23.5%) belong to present limitation of external rotation group, as there was a statistically significant negative relation with *p*-value (*p* < 0.001).

As notices that, the results showed 31 patients out of 70 having posterior extra capsular edema, 21 patients (57.9%) belong to absent limitation of external rotation group and 10 patients (19.6%) belong to present limitation of external rotation group, as there was a statistically significant negative relation with *p*-value (*p* = 0.002).

There is no statistically significant association between absence and presence regarding limitation of ROM “external-rotation” according to obliteration of subcoracoid fat triangle, effusion of biceps tendon sheath, capsular thickening of glenoid portion (mm), capsular thickening of humeral (mm), axillary recess height (mm) and axillary recess width (mm), with *p*-value (*p* > 0.05 NS) (Table 8).

**Table 8:** Association between clinical stage according to MRI finding (*n* = 70)

MRI finding	Stage group			Test value	*p*-value
	Stage 1 (*n* = 17)	Stage 2 (*n* = 27)	Stage 3 and 4 (*n* = 26)		
*Qualitative findings*					
*Capsular edema*					
*Glenoid*					
Absent	8 (47.1%)	14 (51.9%)	21 (80.8%)	*x*^2^: 6.631	0.036*
Present	9 (52.9%)	13 (48.1%)	5 (19.2%)		
*Humeral*					
Absent	8 (47.1%)	10 (37.0%)	21 (80.8%)	*x*^2^: 10.949	0.004*
Present	9 (52.9%)	17 (63.0%)	5 (19.2%)		
*Extra capsular edema*					
*Anterior*					
Absent	10 (58.8%)	14 (51.9%)	21 (80.8%)	*x*^2^: 5.116	0.077
Present	7 (41.2%)	13 (48.1%)	5 (19.2%)		
*Posterior*					
Absent	12 (70.6%)	16 (59.3%)	21 (80.8%)	*x*^2^: 2.922	0.232
Present	5 (29.4%)	11 (40.7%)	5 (19.2%)		
*Obliteration of subcoracoid fat triangle*					
Absent	15 (88.2%)	7 (25.9%)	13 (50.0%)	FE: 16.496	0.002*
Partial obliteration	0 (0.0%)	4 (14.8%)	2 (7.7%)		
Complete obliteration	2 (11.8%)	16 (59.3%)	11 (42.3%)		
*Effusion of biceps tendon sheath*					
Absent	2 (11.8%)	7 (25.9%)	13 (50.0%)	*x*^2^: 7.590	0.022*
Present	15 (88.2%)	20 (74.1%)	13 (50.0%)		
*Quantitative findings*					
*Capsular thickening*					
*Glenoid (mm)*					
Mean ± SD	5.45 ± 1.39	6.04 ± 1.39	5.92 ± 1.04	*F*: 1.187	0.312
Range	2.87–8	3–7.7	4–7.6		
*Humeral (mm)*					
Mean ± SD	5.61 ± 2.00	4.65 ± 1.47	4.27 ± 1.90	*F*: 3.705	0.019*
Range	2.5–8.8	2–7	1.5–9.03		
*Axillary recess*					
*Height (mm)*					
Mean ± SD	7.86 ± 2.21	6.63 ± 2.92	5.64 ± 2.11	*F*: 2.683	0.005*
Range	5.2–11.6	3.49–13.5	3.2–10		
*Width (mm)*					
Mean ± SD	3.05 ± 1.83	2.24 ± 0.94	1.95 ± 0.77	*F*: 2.024	0.019*
Range	1.2–6.8	1.1–3.5	0.9–2.7		

Using: *F*: one way analysis of variance; *x*^2^: Chi-square test; Fisher’s exact test

*p*-value > 0.05 NS; *p-value < 0.05 S

The results showed 27 patients out of 70 having capsular edema of glenoid portion of axillary recess, 9 patients (52.9%) belong to stage 1; 13 patients (48.1%) belong to stage 2 and 5 patients (19.2%) belong to stage 3 + 4, as there was a statistically significant negative relation with *p*-value (*p* = 0.036).

Additionally, the results showed 31 patients out of 70 having capsular edema of humeral portion ofaxillary recess, 9 patients (52.9%) belong to stage 1; 17 patients (63%) belong to stage 2 and 5 patients (19.2%) belong to stage 3 + 4, as there was a statistically significant negative relation with *p*-value (*p* = 0.004).

As well as, the results showed 29 patients out of 70 having obliteration of subcoracoid fat triangle, 2 patients (11.8%) belong to stage 1; 16 patients (59.3%) belong to stage 2 and 11 patients (42.3%) belong to stage 3 + 4, as there was a statistically significant relation with *p*-value (*p* = 0.002).

As notices that, the results showed 48 patients out of 70 having effusion of biceps tendon sheath, 15 patients (88.2%) belong to stage 1; 20 patients (74.1%) belong to stage 2 and 13 patients (50%) belong to stage 3 + 4, as there was a statistically significant negative relation with *p*-value (*p* = 0.022).

It is clear that, there was a statistically significant difference between stage group according to capsular thickening of humeral portion of axillary recess (mm), with *p*-value (*p* < 0.05). The highest value was found in stage 1 (5.61 ± 2.00), followed by stage 2 (4.65 ± 1.47) and the lowest value was found in stage 3 + 4 (4.27 ± 1.90).

Whenever, there was a statistically significant difference between stage group according to axillary recess height (mm), with *p*-value (*p* < 0.05). The highest value was found in stage 2 (7.86 ± 2.21), followed by stage 1 (6.63 ± 2.92) and the lowest value was found in stage 3 + 4 (5.64 ± 2.11).

Also, there was a statistically significant difference between stage group according to capsular thickening of humeral portion of axillary recess (mm), with *p*-value (*p* < 0.05). The highest value was found in stage 1 (5.61 ± 2.00), followed by stage 2 (4.65 ± 1.47) and the lowest value was found in stage 3 + 4 (4.27 ± 1.90).

While, there was a statistically significant difference between stage group according to axillary recess width (mm), with *p*-value (*p* < 0.05). The highest value was found in stage 1 (3.05 ± 1.83), followed by stage 2 (2.24 ± 0.94) and the lowest value was found in stage 3 + 4 (1.95 ± 0.77).

There is no statistically significant difference between clinical stage according to anterior extra capsular edema, posterior extra capsular edema and capsular thickening of glenoid portion of axillary recess (mm), with *p*-value (*p* > 0.05 NS).

Table 9 shows statistically significant positive correlation between pain intensity with capsular thickening of humeral portion of axillary recess and statistically significant negative correlation with axillary recess height “mm”, while the rest have insignificant correlation.

**Table 9:** Results of linear regression analysis to estimate variables which are the most predictive of clinical impairment at pain intensity

MRI finding	*β*	Odds ratio	95% CI		*p*-value
			Lower	Upper	
*Qualitative findings*					
*Capsular edema*					
Glenoid	5.924	1.594	1.518	1.673	0.071
Humeral	8.080	3.098	1.816	5.283	0.146
*Extra capsular edema*					
Anterior	6.094	2.009	1.557	2.589	0.139
Posterior	2.714	0.778	0.153	1.791	0.224
*Obliteration of subcoracoid fat triangle*	− 8.614	0.562	0.227	1.394	0.101
*Effusion of biceps tendon sheath*	− 1.519	0.732	0.144	1.685	0.649
*Quantitative findings*					
*Capsular thickening*					
Glenoid (mm)	− 8.664	0.755	0.149	1.737	0.381
Humeral (mm)	14.598	4.901	2.233	13.303	0.028*
*Axillary recess*					
Height (mm)	− 13.965	2.232	1.669	4.986	0.015*
Width (mm)	− 3.390	1.736	1.555	1.937	0.276

*β*: regression coefficient, OR: odds ratio, CI: confidence interval

Table 10 shows statistically significant negative correlation between limitation of ROM “abduction” with anterior extra capsular edema, while the rest have insignificant correlation.

**Table 10:** Results of linear regression analysis to estimate variables which are most predictive of clinical impairment at limitation of ROM “abduction”

MRI finding	*β*	Odds ratio	95% CI		*p*-value
			Lower	Upper	
*Qualitative findings*					
*Capsular edema*					
Glenoid	− 2.666	1.985	1.060	4.484	0.085
Humeral	3.988	1.123	0.765	3.004	0.129
*Extra capsular edema*					
Anterior	− 17.024	4.653	2.846	8.774	0.017*
Posterior	− 5.027	1.455	1.385	1.527	0.351
*Obliteration of subcoracoid fat triangle*	− 4.273	1.971	1.061	2.669	0.082
*Effusion of biceps tendon sheath*	− 1.430	2.301	1.721	3.079	0.090
*Quantitative findings*					
*Capsular thickening*					
Glenoid (mm)	3.110	1.684	1.508	1.879	0.469
Humeral (mm)	− 3.588	0.689	0.136	1.585	0.874
*Axillary recess*					
Height (mm)	2.221	1.369	1.030	1.877	0.138
Width (mm)	1.426	0.668	0.132	1.538	0.186

*β*: regression coefficient, OR: odds ratio, CI: confidence interval

Table 11 shows statistically significant negative correlation between limitation of ROM at “external rotation” with capsular edema of humeral portion of axillary recess and anterior extra capsular edema, while the rest have insignificant correlation.

**Table 11:** Results of linear regression analysis to estimate variables which are most predictive of clinical impairment at limitation of ROM “external rotation”

MRI finding	*β*	Odds ratio	95% CI		*p*-value
			Lower	Upper	
*Qualitative findings*					
*Capsular edema*					
Glenoid	− 2.906	0.513	0.207	1.273	0.497
Humeral	− 16.766	6.909	3.653	9.403	0.042*
*Extra capsular edema*					
Anterior	− 11.766	4.343	3.882	7.622	0.017*
Posterior	− 2.118	2.523	1.761	4.124	0.078
*Obliteration of subcoracoid fat triangle*	− 1.955	1.949	1.151	2.512	0.236
*Effusion of biceps tendon sheath*	5.344	2.165	1.619	2.897	0.259
*Quantitative findings*					
*Capsular thickening*					
Glenoid (mm)	− 0.612	1.754	0.657	5.904	0.483
Humeral (mm)	− 6.361	0.529	0.213	1.312	0.292
*Axillary recess*					
Height (mm)	3.631	2.702	1.543	8.821	0.352
Width (mm)	1.684	1.890	1.465	2.436	0.401

*β*: regression coefficient, OR: odds ratio, CI: confidence interval

### Sample of study cases

**Fig. 1:**
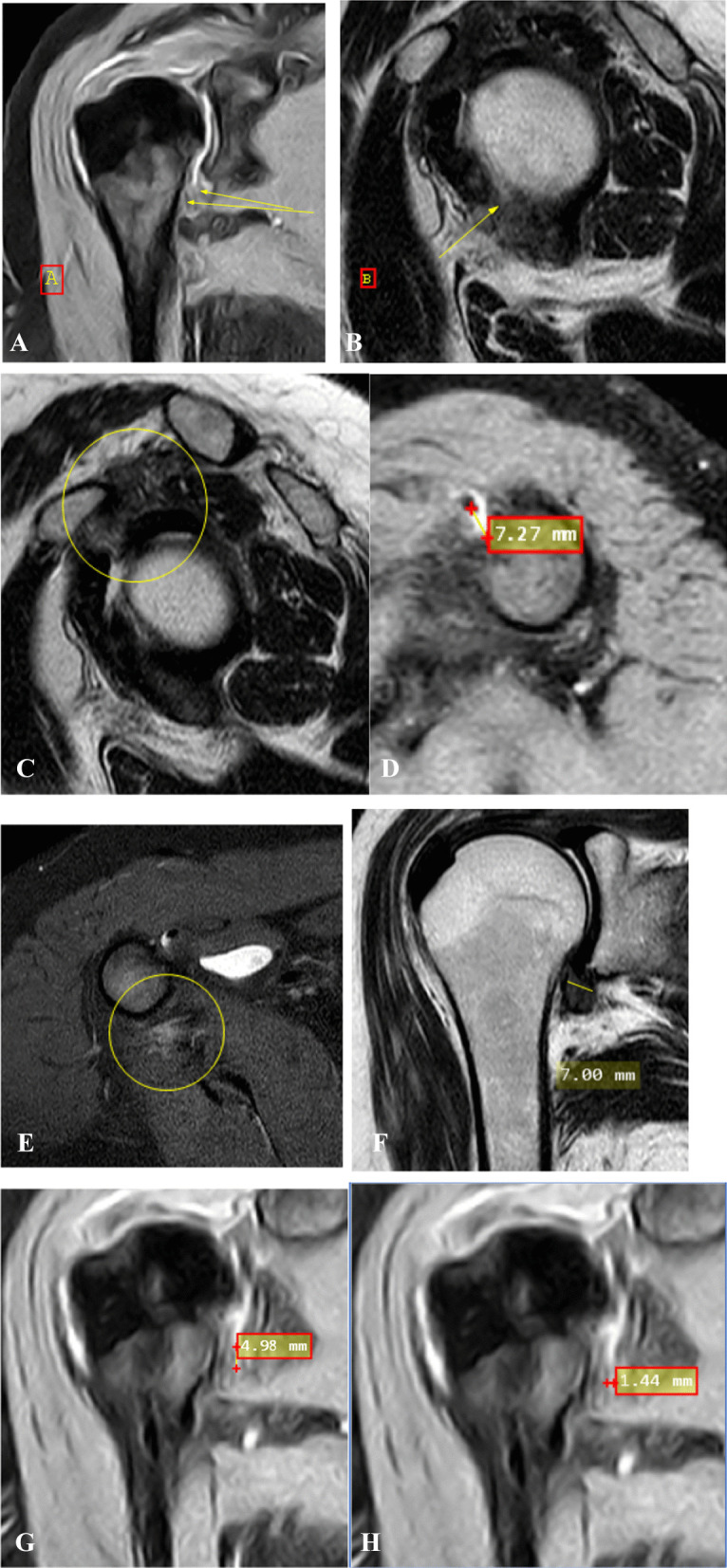
**A** Coronal fat-suppressed PD-weighted MRI image shows capsular edema at glenoid and humeral portions of capsule (arrows). **B** Sagittal T2-weighted MRI image shows anterior extracapsular edema (arrow). **C** Sagittal T2-weighted MRI image shows complete obliteration of subcoracoid fat triangle (encircled area). **D** Axial fat-suppressed PD MRI image shows effusion at long head biceps tendon sheath more than 2 mm (arrow). **E** Axial fat-suppressed PD-weighted MRI image shows extracapsular edema (encircled area). **F** Coronal T2-weighted MRI image shows maximum capsular thickening at glenoid portion of axillary recess, the humeral portion was obliterated and fibrotic. **G**, **H** Coronal fat-suppressed PD-weighted MRI image shows measurement of maximal height and width of axillary recess

Case 1: A case of 54 years, female, clinical presentation: minimal pain radiating to whole right limb with no limitation of ROM of 5 months duration. Pain score: 1. Clinical stage: stage 2 (acute) (Fig. 1).

**Fig. 2:**
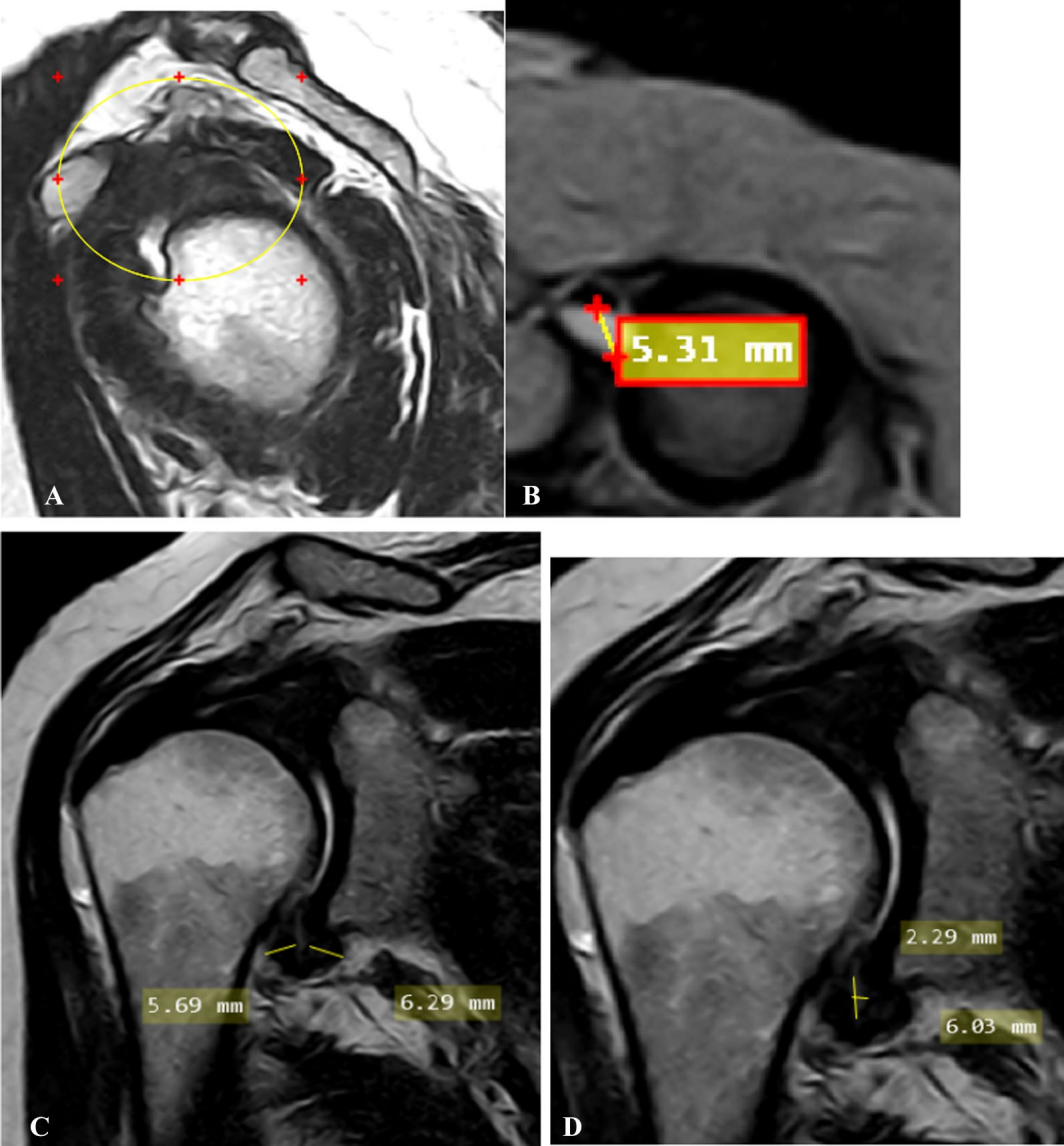
**A** Oblique sagittal T2-weighted MRI image shows complete obliteration of subcoracoid fat triangle (encircled area). **B** Oblique axial fat- suppressed PD weighted MRI image shows effusion at long head biceps tendon sheath more than 2mm. **C** Oblique coronal T2-weighted MR image shows measurement of thickest portion of joint capsule in humeral and glenoid portions of axillary recess. **D** Oblique coronal T2-weighted MR image shows measurement of maximal height and width of axillary recess

Case 2: A case of female patient has 57 years old with left shoulder minimal pain with limitation of ROM on both abduction and external rotation of 9 months duration. Pain score on pain scale: 2. Clinical stage: 2 (subacute) (Fig. 2).

**Fig. 3:**
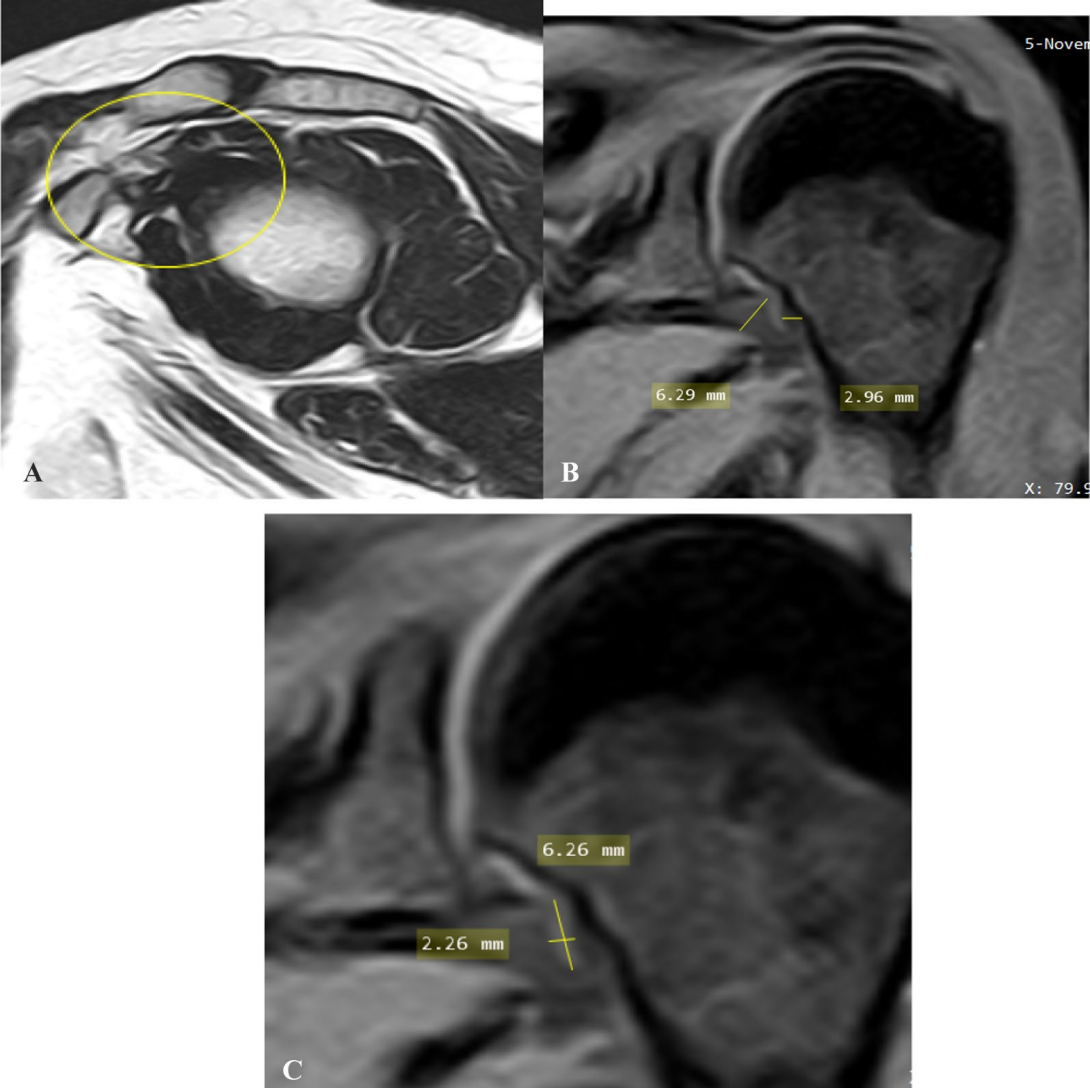
**A** Oblique sagittal T2-weighted MR image shows partial obliteration of subcoracoid fat triangle with no observed fat planes or coracohumeral ligament (encircled area). **B** Oblique coronal fat-suppressed PD-weighted MR image shows maximum capsule thickness at glenoid and humeral portions of axillary recess, the humeral was fibrotic and obliterated. **C** Oblique coronal fat-suppressed PD-weighted MR image shows measurement of maximal height and width of axillary recess

Case 3: A case of female patient has 47 years old with pain radiating to the whole left limb in both state and movement of mild degree with limitation of ROM of both abduction and external rotation of 1 year duration. Pain score on pain scale: 3. Clinical stage: 3 (subacute to chronic) (Fig. 3).

**Fig. 4:**
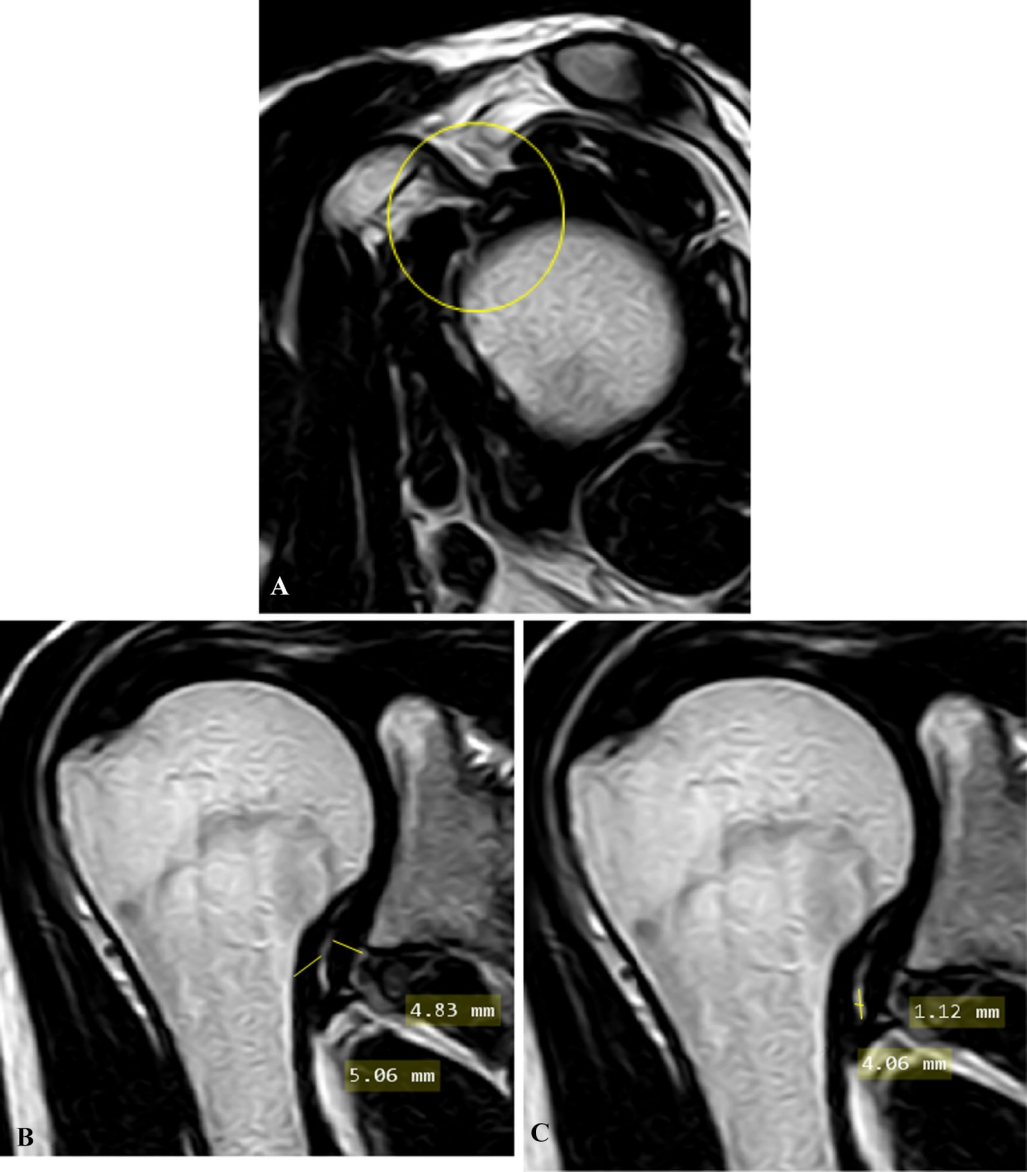
**A** Oblique sagittal T2-weihted MR image shows preserved fat planes and coracohumeral ligament of the rotator interval (encircled area). **B** Oblique coronal T2-weighted MR image shows thickened joint capsule and measurement of maximum capsular thickness of glenoid and humeral portions of axillary recess. **C** Oblique coronal T2-weighted MR image shows measurement of maximum height and width of axillary recess at the same plan, it was fibrotic and obliterated

Case 4: A case of male patient has 31 years old with limitation of ROM on abduction only, yet no pain of 7 years duration. Pain score on pain scale: 0. Clinical stage: 4 (chronic) (Fig. 4).

**Fig. 5:**
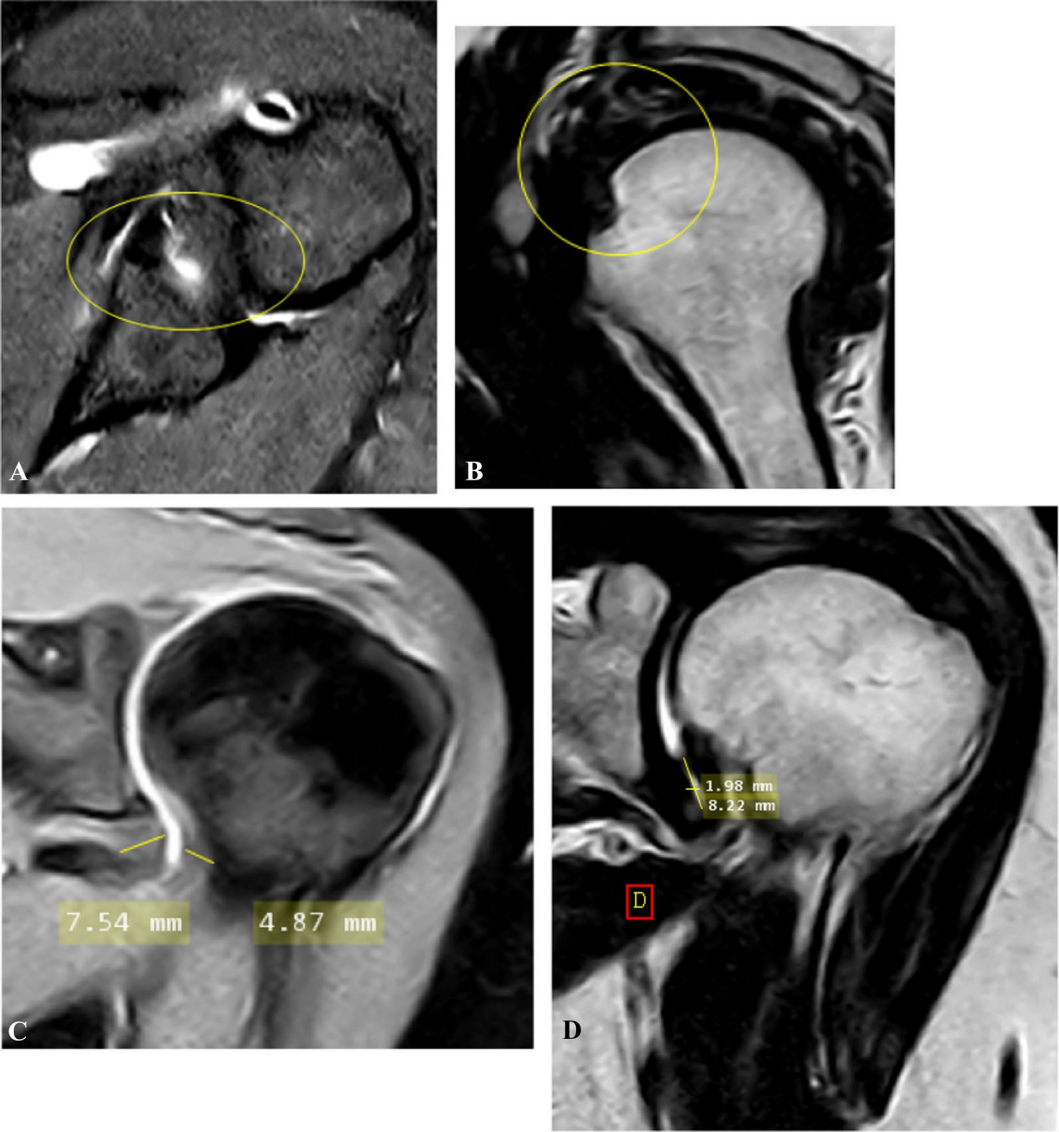
**A** Oblique axial fat-suppressed PD-weightrd MR image shows anterior and posterior extracapsular edema (hyperintensity) (encircled area). **B** Oblique sagittal T2-weighted MR image shows partial obliteration of subcoracoid fat triangle (encircled area). **C** Oblique coronal fat-suppressed PD-weighted MR image shows measurement of thickest portion of joint capsule in humeral and glenoid portions of axillary recess. **D** Oblique coronal T2-weighted MR image shows measurement of maximal height and width of axillary recess

Case 5: A case of female patient has 51 years old with: moderate left shoulder pain and limitation of ROM on external rotation only of two months duration, Pain score on pain scale: 5. Clinical stage: 2 (subacute) (Fig. 5).

**Fig. 6:**
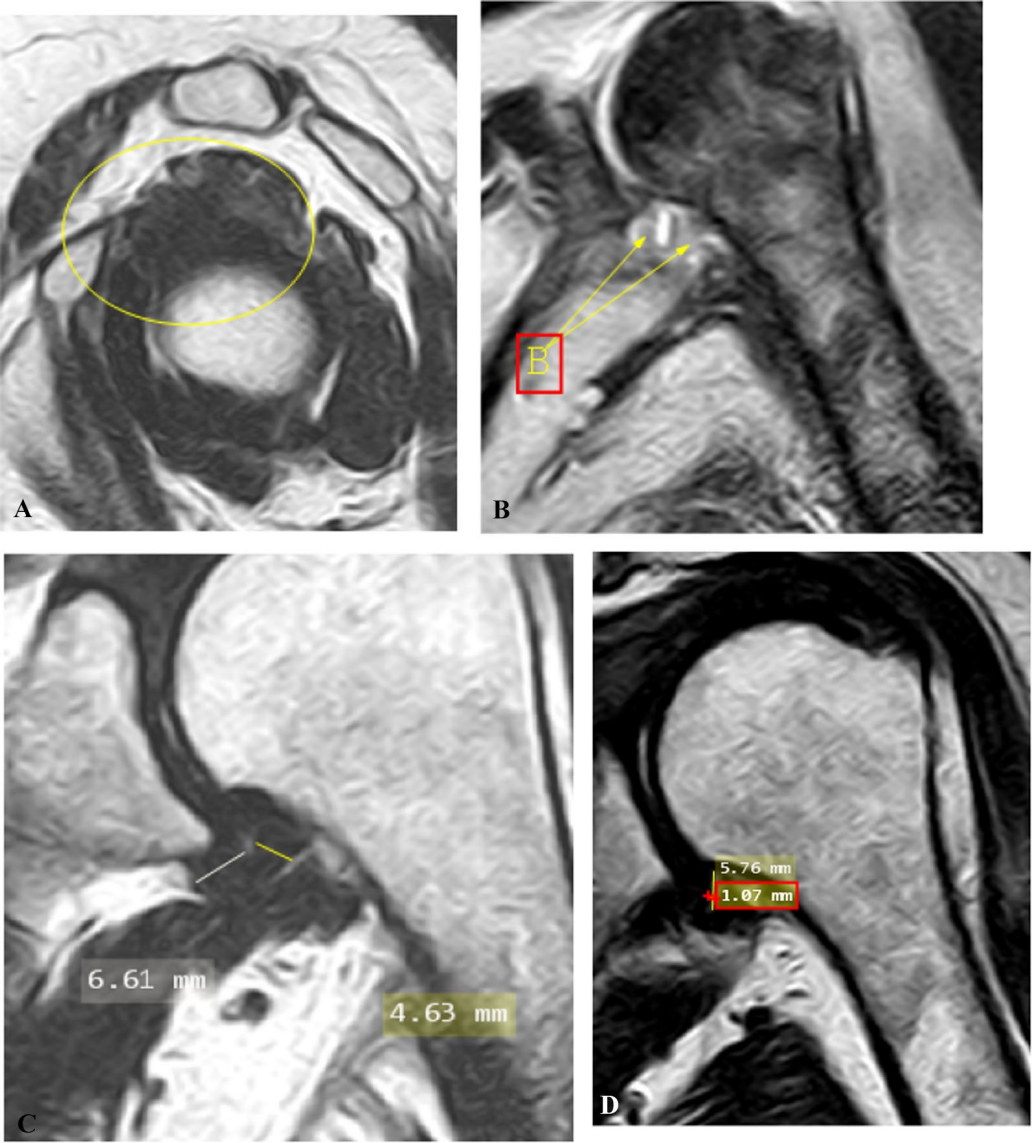
**A** Oblique sagittal T2-weighted MR image shows complete obliteration of subcoracoid fat triangle with no observed fat planes or coracohumeral ligament (encircled area). **B** Oblique coronal fat-suppressed PD-weighted MR image shows edema at glenoid and humeral portions of axillary recess (arrows). **C** Oblique coronal T2-weighted MR image shows measurement of thickest portion of joint capsule in humeral and glenoid portions of axillary recess. **D** Oblique coronal T2-weight MR image shows measurement of maximal height and width of axillary recess

Case 6: A case of female patient had 50 years old with severe left shoulder pain, yet no limitation of ROM of three months of duration. Pain score on pain scale: 7. Clinical stage: stage 1 (acute) (Fig. 6).

**Fig. 7:**
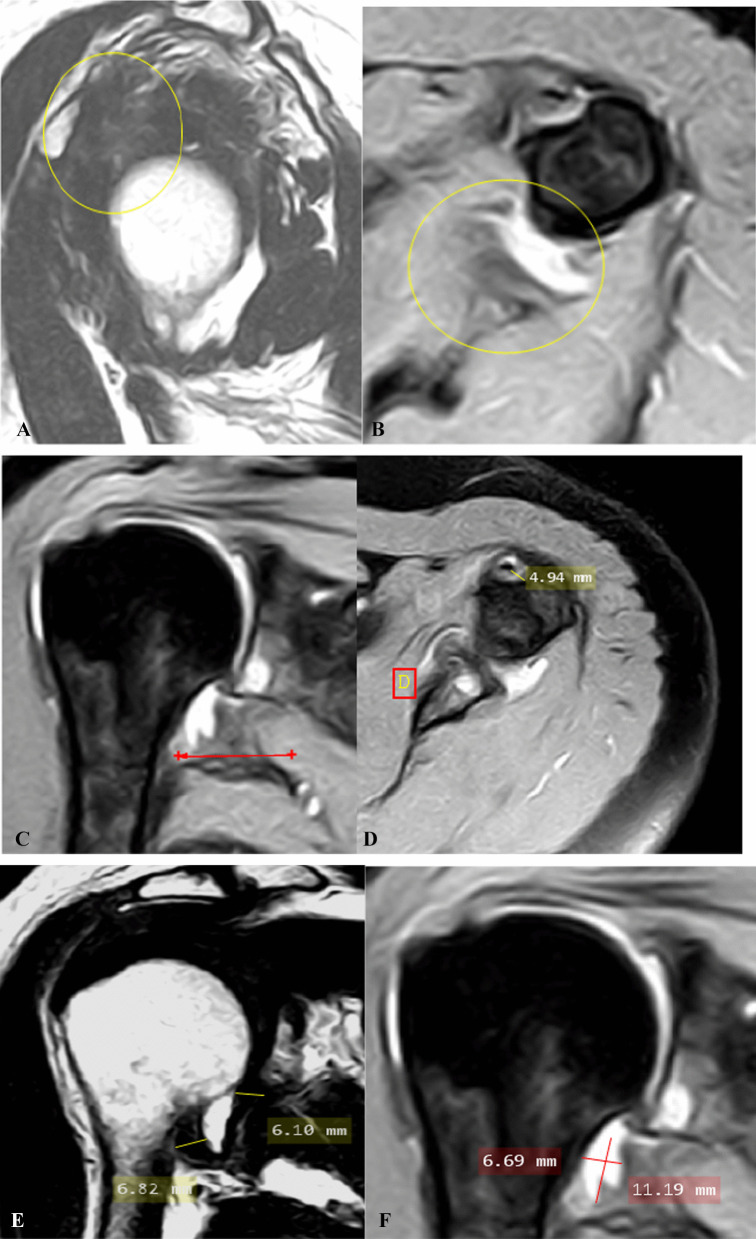
**A** Oblique sagittal PD- weighted MR image shows complete obliteration of the rotator interval with no observed fat planes or coracohumeral ligament (encircled area). **B** Oblique axial fat-suppressed PD-weighted MR image shows anterior and posterior extracapsular edema (encircled area). **C** Oblique coronal fat-suppressed PD-weighted MR image shows edema at humeral portion of axillary recess (arrow). **D** Oblique axial fat-suppressed PD weighted MR image shows effusion in long head biceps tendon sheath (arrow). **E** Oblique coronal T2-weighted MR image shows thickened joint capsule and measurement of maximum capsular thickness of glenoid and humeral portions of axillary recess with effusion at axillary recess. **F** Oblique coronal fat-suppressed PD-weighted MR image shows measurement of maximum height and width of axillary recess at the same plan

Case 7: A case of female patient has 62 years old with moderate left shoulder pain of sudden onset and progressive course with neck stiffness with limitation of ROM on both abduction and external rotation. Duration of symptoms: 3 months. Pain score on pain scale: 6. Clinical stage: 2 (acute) (Fig. 7).

## Discussion

Adhesive capsulitis, also called frozen shoulder is a clinical syndrome of pain and severely decreased joint motion caused by thickening and contraction of the joint capsule and synovium. The risk factors are female sex, patients aged > 40 years, trauma, diabetes, prolonged immobilization, thyroid disease, stroke, myocardial infarction, and autoimmune disease [19].

Although spontaneous resolution is the rule, years can ensue (mean 18–30 months) before joint mobility returns to normal. Various treatment options exist for AC (e.g., oral anti-inflammatory drugs, intraarticular corticoid injection, physiotherapy, percutaneous capsular distention, surgical release, etc.) depending on the level of clinical impairment, and on an accurate diagnosis. Thus, disease staging and identification of inflammatory changes could have an impact on patient management [20].

The diagnosis of AC might be challenging because it is a diagnosis of exclusion, and it is mainly based on the clinical examination, with the only additional study suggested being the plain shoulder radiograph to rule out other possible causes of a limited ROM such as osteoarthritis, fracture, and chronic shoulder dislocation [13].

Recent studies have shown that Magnetic Resonance Imaging (MRI) can provide reliable imaging indicators of frozen shoulder. Potentially useful MR findings in frozen shoulder include thickening of the CHL, thickening of the joint capsule in the rotator cuff interval, and obliteration of the fat triangle under the coracoid process [4].

The aim of work of the current study was to examine the association between clinical features (stages) and MRI findings in adhesive capsulitis of the shoulder. To elucidate the aim of this study, 70 patients were included. In the present study,there was female predominance (62.9%) with female to male ratio about 1.7:1 which agree with Park et al. [5] who found that among the 103 patients there were 62 women, 41 men. That also, agree with Ewald [6] who stated that women are more often affected than men.

In the current study, the mean age of patients with AC is 44.84 ± 10.65 years ranged between 22 to 62 years which near to the results in the study done by Park et al.[5] who found that mean age, 51.9 years; range, 34–75 years. Also, in the study done by Pag [7] the mean age of the studied cases was 54.1 years ranged between 22 and 78 years.

In this study, the duration of symptoms “months” ranged between 0.08–7 months with mean 0.86 ± 1.36 months but in the study by Park et al. [5] who found that the duration of symptoms at the time of medical record analysis varied from 2 to 24 months (mean, 5.8 months).

As regard to the affected side, there were 35 patients (50.0%) had the right side of shoulder and 35 patients (50.0%) had left side of shoulder affection which near to the results in the study done by Do et al. [8] who found that 28 patients (45.9) had right shoulder affected and 33 patients have left side shoulder affection (54.1).

Although various structural abnormalities have been associated with adhesive capsulitis, only a few studies have examined the association between MRI findings and clinical features [8–11].

Teixeira et al. [12] reported that edema at the axillary recess is a good diagnostic indicator with excellent reproducibility and high sensitivity. In line with this conclusion, we found that joint capsule edema in the humeral portion of the axillary recess on fat suppressed T2-weighted MR images was present in 44.3% of adhesive capsulitis patients in our study and in the glenoid portion in 38.6% while there were 25 patients (35.7%) were anterior and 21 patients (30.0%) were posterior among extra capsular edema. Teixeira et al. [12] also suggested that a layer of high signal intensity bordering the outer capsular surface of the shoulder joint on fat-suppressed T2-weighted MR images is highly specific for the diagnosis of adhesive capsulitis, consistent with the MRI findings of extracapsular edema in our study.

As regard to stages; there were 17 patients (24.3%) were stage 1, 27 patients (38.6%) were stage 2, 21 patients (30.0%) were stage 3 and 5 patients (7.1%) were stage 4 among Stage while in the study done by Park et al. [5], thirty-eight patients had stage 1 capsulitis; 52 patients, stage 2; and 14 patients, stages 3 and 4.

Although the pathophysiologic mechanism of adhesive capsulitis is controversial, pathologic findings reflect both synovial inflammatory and capsular fibrotic conditions, depending on the clinical stage [13]. The early stages of adhesive capsulitis involve considerable pain with gradually increasing joint stiffness caused by ongoing synovial inflammation and fibrosis. During the later stages, as inflammation decreases, capsular fibrosis reaches its peak [14]. Several studies have shown that gadolinium enhancement of the joint capsule and synovial membrane is common in stage 2 adhesive capsulitis [15, 16].

In the present study, obliteration of subcoracoid fat triangle was detected in 35 patients (50%) moreover, in 17 of 22 patients, Mengiardi et al. [17] found an inflammatory obliteration of the subcoracoid fat triangle with AC, which we also observed in partial or complete form in this investigation. The complete obliteration of this fat triangle was specific to the diagnosis of frozen shoulder or AC.

In our study, the mean capsular thickening of glenoid portion of axillary recess (mm) (5.85 ± 1.28) and the mean thickening of humeral portion of axillary recess (mm) (5.11 ± 1.79) which is near to the results in the study done by Park et al. [5] who found that joint capsule thickness in the axillary recess was 4.06 ± (SD) 1.59 mm in the humeral portion and 4.34 ± 1.46 mm in the glenoid portion. Capsule thickness in the axillary recess has been described as a reliable diagnostic tool of AC when > 4 mm [17, 18].

In this study, joint capsule thickness in the humeral portion of the axillary recess and pain intensity had a positive correlation (*p* = 0.046. There was a negative correlation between height of the axillary recess and pain intensity (*p* = 0.033).In the current study, we found statistically significant negative linear correlations between anterior extracapsular edema and ROM on abduction with (*p* = 0.014. In the present study, we found statistically significant negative linear correlations between anterior extracapsular edema and limited ROM on external rotation (*p* = 0.001). In our study, there was statistically significant negative linear correlations between joint capsule edema in the humeral portion of the axillary recess and limited ROM on external rotation (*p* = 0.013 and all of theses results previously mentioned coincide with Park et al. [5].

That means, patients with limited ROM on external rotation and abduction had anterior extracapsular edema and joint capsule edema in the humeral portion of the axillary recess on fat-suppressed T2-weighted MR images. In the present study, there were no significant correlations between obliteration of the subcoracoid fat triangle, effusion in the long head biceps tendon sheath, joint capsule thickness in the humeral portion of the axillary recess, width of the axillary recess, or degree of external rotation during MRI (*p* > 0.05) which also are in agreement with Park et al. [5].

In this study, no statistically significant association between absence and presence of limitation of ROM “external-rotation” according to capsular thickening of glenoid portion of axillary recess (mm), capsular thickening of humeral portion of axillary recess (mm) which is disagree with Ahn et al. [10] who found a significant negative correlation between the limitation of external rotation and axillary joint capsule thickness.

In the current study, effusion in the long head biceps tendon sheath was not significantly correlated with pain intensity, limitation of ROM on external rotation and ROM on abduction which agree with Park et al. [5] who stated that effusion in the long head biceps tendon sheath was not accompanied by fluid in the shoulder joint capsule and was not significantly correlated with clinical features.

In the present study, There is no statistically significant association between absence and presence of limitation of ROM “external-rotation” according to capsular thickening of glenoid portion of axillary recess (mm) and capsular thickening of humeral portion of axillary recess (mm) which agree with Lee et al. [11] who reported no significant correlation between joint capsule thickness in the axillary recess with either limitation of ROM on external rotation or abduction.

But that disagree with Ahn et al. [10] who found that joint capsule thickness in the axillary recess significantly correlated with ROM on external rotation in patients with adhesive capsulitis, but this finding was not associated with abduction ROM or pain intensity. This discrepancy may be related to differences in measurement methods.

In our study, thicknesses of the joint capsule in the humeral and glenoid portions of the axillary recess were measured separately and analyzed, whereas in previous studies, joint capsule thickness was measured in only one location. In addition, one of the previous studies was conducted with direct MR arthrography Lee et al. [11].

Regarding to capsular edema of humeral portion of axillary recess, the results showed 31 patients out of 70 having capsular edema of humeral portion of axillary recess, 9 patients (52.9%) belong to stage 1; 17 patients (63%) belong to stage 2 and 5 patients (19.2%) belong to stage 3 + 4 while in the study done by Park et al. [5]. Joint capsule edema in the humeral portion of the axillary recess was found in 97% of patients with stage 1 capsulitis, in 83% with stage 2, and in 64% with stages 3 and 4.

In the study done by Chellathurai et al. [19], edema on the humeral aspect was present in 100% of the patients in stage I adhesive capsulitis, 88.5% in stage II capsulitis, 5% in stage III capsulitis, and 0% in stage IV. Mild edema was present in 14.3% in stage III and 7.7% in stage IV. The distribution of edema in the different clinical stages was significant (*P* 0.001).

In the current study, joint capsule edema in the humeral portion of the axillary recess was more common in patients with stage 2 and that of glenoid portion is more common in stage 1, also there was a statistically significant difference between stage group according to capsular thickening of humeral portion of axillary recess (mm) which is in agreement with Sofka et al. [8] who found that joint capsule thickness in the axillary recess correlates with clinical stage and that joint capsule edema in the axillary recess is common in stage 2.

In the study done by Chellathurai et al. [19] the incidence of edema was more common in stages I and II which progressively decreases in stages III and IV.

In our study, 29 patients out of 70 having obliteration of subcoracoid fat triangle, 2 patients (11.8%) belong to stage 1; 16 patients (59.3%) belong to stage 2 and 11 patients (42.3%) belong to stage 3 + 4, as there was a statistically significant relation. Ahn et al. [10], Lee et al. [11] reported no association between obliteration of the subcoracoid fat triangle and clinical impairment. Park et al. [5] confirmed this lack of association, obliteration of the subcoracoid fat triangle was seen more frequently during the early (stages 1 and 2) than later (stages 3 and 4) stages of adhesive capsulitis. Obliteration of the subcoracoid fat triangle may thus be related to inflammation, which is frequently extensive during the early stages of adhesive capsulitis.

Chellathurai et al. [19] stated that obliteration of the fat in the subcoracoid triangle was present in 44.4% in stage I, 46.2% in stage II, 90.5% in stage III, and 84.6% in stage IV. This distribution was significant (P 0.005).

As regard to effusion of long head biceps tendon sheath, the results showed 48 patients out of 70 having effusion of biceps tendon sheath, 15 patients (88.2%) belong to stage 1; 20 patients (74.1%) belong to stage 2 and 13 patients (50%) belong to stage 3 + 4, as there was a statistically significant negative relation with *p*-value (*p* = 0.022) while in the study done by Chellathurai et al. [19] effusion around the long head biceps tendon was found in 100% in stage I, 96.2% in stage II, 81% in stage III, and 61.5% in stage IV. The highest incidence was found in stage I, than in stage II, then decreases in stages III and IV. This distribution was statistically significant (*P* 0.016).

### Study limitations

Our study was limited by that the most of patients had a limitation of ROM on external rotation, so we could not measure the degree of external rotation during the MRI scan. The clinical information was not sufficient to correlate between the clinical features and MRI findings.

The relatively long period of the lockdown for COVID-19 pandemic with its negative impact on the flow of cases.

We did not use the arthroscopy as a gold standard for this study except for examination the pathophysiology of AC.

## Conclusions

Adhesive capsulitis (AC) of the shoulder is a common condition with an incidence in the general population varying considerably from 2 to 5.3% for primary and from 4.3 to 38% for secondary AC. Although spontaneous resolution is the rule, years can ensue (mean 18–30 months) before joint mobility returns to normal.

It is characterized by gradual and progressive onset of shoulder pain and limited active and passive range of motion (ROM) in the shoulder. in the past the diagnostic terminology for this entiry, such as "frozen shoulder", was ambigious and based on clinical features and symptoms, medical history, and physical examination. However, the disease presents with characteristic pathophysiology features, including capsular thickening and fibrosis due to chronic inflammation of the joint capsule, which may lead to capsular adhesion.

Based on previous studies that used MRI, the key diagnostic findings for AC include capsular thickening, a hyperintense T2 signal and contrast enhancement in the axillary capsule and rotator interval, and obliteration of the subcoracoid fat triange. Theses MRI findings have an important role in the diagnosis of early AC.

## Data Availability

The data is available at the editorial board’s request.

## Abbreviations

CHL: Coracohumeral
MRI: Magnetic resonance imaging
SD: Standard deviation
SPSS: Statistical Package for Social Science
AC: Adhesive capsulitis
ROM: Range of motion
